# S76. Adoptive cell therapy of melanoma with autologous tumour infiltrating lymphocytes

**DOI:** 10.1186/2051-1426-2-S2-I14

**Published:** 2014-03-12

**Authors:** I Svane, R Andersen, M Donia

**Affiliations:** 1 Copenhagen University Hospital, Department of Oncology Center for Cancer Immune Therapy, Herlev, Denmark

## Background

Adoptive T-cell therapy (ACT) with tumor infiltrating lymphocytes (TILs) is a personalized treatment for cancer defined as the infusion of T cells isolated from the patient’s own tumor tissue after ex vivo activation and several rounds of expansion. This treatment has achieved impressive clinical results in several single institution phase I/II clinical trials performed outside Europe, and holds the promise to enter the mainstream of standard melanoma care in the near future. However, although transient, the toxicities associated with high-dose IL-2 classically administered together with TILs are severe and recent data have questioned its use.

## Material and methods

In an ongoing phase I/II study, we have enrolled patients with progressive metastatic melanoma. TILs infusion was preceded by standard lymphodepleting chemotherapy but followed by low-dose subcutaneous IL-2 for 14 days or by an intravenous intermediate dose IL-2 decrescendo regimen.

## Results

The lower doses of IL-2 considerably decreased the toxicity of the treatment while PET-CT imaging showed a preserved objective response rate of 48% including long-lasting complete responders. The absolute number of tumour specific T-cells infused was significantly associated to clinical response, with induction of peripheral tumour reactive T cells. To characterize the fate of TILs after infusion, we performed a longitudinal analysis of bulk tumor-reactive T cells from in vitro cultured TILs and found durable persistent T-cells after up to 1.5 years after infusion. Extensive analysis of disease relapse in two patients revealed multiple potential mechanisms of tumor recurrence, linked either to tumor-changes or immune response decline.

## Conclusion

Despite its clinical efficacy, with impressive response rates and a significant fraction of long surviving complete responders, the implementation of TIL based ACT into current practice has been severely hampered by the technical complexity of cell production, the toxicity profile demanding treatment at specialize cancer centers, and lack of investment from the pharmaceutical industry. Next step should be a pivotal phase III trial in melanoma which is required for regulatory approval. Further improvement of the therapy could also be pursued through combination treatment.

